# Immunotherapy Approaches in Isocitrate-Dehydrogenase-Mutant Low-Grade Glioma

**DOI:** 10.3390/cancers15143726

**Published:** 2023-07-22

**Authors:** Marco Gallus, Darwin Kwok, Senthilnath Lakshmanachetty, Akane Yamamichi, Hideho Okada

**Affiliations:** 1 Department of Neurological Surgery, University of California, San Francisco, CA 94143, USA; marco.gallus@ucsf.edu (M.G.); darwin.kwok@ucsf.edu (D.K.); senthilnath.lakshmanachetty@ucsf.edu (S.L.); akane.yamamichi@ucsf.edu (A.Y.); 2 Department of Neurosurgery, University Hospital Muenster, 48149 Muenster, Germany; 3 Parker Institute for Cancer Immunotherapy, San Francisco, CA 94129, USA; 4 Helen Diller Family Comprehensive Cancer Center, San Francisco, CA 94143, USA

**Keywords:** low-grade glioma, immunotherapy for glioma, IDH-mutant glioma, glioma vaccine, IDH-1 mutant inhibitor, low intensity-focused ultrasound, antigen heterogeneity

## Abstract

**Simple Summary:**

IDH-mutant low-grade gliomas (LGG) are slow-growing glial cell-derived tumors of the central nervous system (CNS) that predominantly manifest in young adults and often show malignant transformation. Despite the therapeutic advances in several other oncologic areas, they are still considered incurable. Immunotherapies offer new therapeutic opportunities; however, they remain ineffective in treating LGG patients. In this review, we aim to summarize the relevant preclinical and clinical research findings and discuss the challenges and lessons learned from those trials. Furthermore, future perspectives on improving the efficacy of immunotherapy for IDH-mutant LGG are highlighted.

**Abstract:**

Low-grade gliomas (LGGs) are slow-growing tumors in the central nervous system (CNS). Patients characteristically show the onset of seizures or neurological deficits due to the predominant LGG location in high-functional brain areas. As a molecular hallmark, LGGs display mutations in the isocitrate dehydrogenase (IDH) enzymes, resulting in an altered cellular energy metabolism and the production of the oncometabolite D-2-hydroxyglutarate. Despite the remarkable progress in improving the extent of resection and adjuvant radiotherapy and chemotherapy, LGG remains incurable, and secondary malignant transformation is often observed. Therefore, novel therapeutic approaches are urgently needed. In recent years, immunotherapeutic strategies have led to tremendous success in various cancer types, but the effect of immunotherapy against glioma has been limited due to several challenges, such as tumor heterogeneity and the immunologically “cold” tumor microenvironment. Nevertheless, recent preclinical and clinical findings from immunotherapy trials are encouraging and offer a glimmer of hope for treating IDH-mutant LGG patients. Here, we aim to review the lessons learned from trials involving vaccines, T-cell therapies, and IDH-mutant inhibitors and discuss future approaches to enhance the efficacy of immunotherapies in IDH-mutant LGG.

## 1. Introduction

Immunotherapies have achieved significant success in various cancer types, such as lung, skin, colon, and hematopoietic malignancies [1,2]. Hence, in recent years, scientists have been striving to apply these achievements to combat brain tumors that have proven to be untreatable thus far. A plethora of preclinical and clinical trials have been conducted to target malignant high-grade gliomas (HGGs), yielding limited success. The endeavors have been extensively reviewed [3,4,5,6,7]. Among other factors, the development of immunotherapies for HGGs poses challenges due to the older age range of patients affected, with the peak incidence of the disease occurring between 75 and 84 years of age, and the fact that HGGs show a highly immunosuppressive tumor microenvironment and rapid progression [8]. 

In contrast, low-grade gliomas (LGGs) exhibit substantially slower tumor growth and commonly manifest in younger patients, predominantly between their 3rd and 4th lives [9]. Taking this into consideration, it appears reasonable to conjecture whether immunotherapies may be more effective in LGG compared to HGG. Younger patients might have a healthier immune system, which allows for multiple treatments that are necessary to mount robust immune responses. Furthermore, targeting HGG-related antigens in LGG may prevent recurrence and secondary transformation into HGG. 

In this review, we highlight challenges and lessons learned from past immunotherapeutic approaches targeting IDH-mutant LGG (Table 1) and outline future perspectives to promote the efficacy of immunotherapy in LGG.

## 2. IDH-Mutant Low-Grade Glioma

According to the most recently published WHO classification of 2021, LGG are characterized by mutations in the isocitrate dehydrogenase (IDH) 1 and IDH 2 genes. Consequently, previously described low-grade gliomas without the mutant IDH, such as the IDH-wildtype diffuse astrocytoma, are now classified as molecular high-grade gliomas [10,11]. Furthermore, in contrast to the previous WHO classification, this novel guideline now summarizes astrocytoma and oligodendroglioma (which also harbor 1p/19q-codeletion) as low-grade gliomas [10,11].

LGG typically become apparent with the onset of new seizures or new focal neurologic deficits, as LGGs are often localized in high-functioning brain areas such as the insula and the supplementary motor cortex [12].

Currently, the standard of care for IDH-mutant LGG involves complete and safe maximal resection to procure tissue for detailed histological, genetic, and molecular analysis [9]. Surgery is followed by (delayed) adjuvant radiotherapy (RT) combined with chemotherapy, either with temozolomide or procarbazine, lomustine, and vincristine (PCV) [13]. Magnetic resonance imaging (MRI) surveillance without any adjuvant therapy is only recommended in young patients (age under 40) with oligodendroglioma that underwent gross total resection [13]. Despite the recent revision of the World Health Organization (WHO) classification and the requirement for further studies to examine the prognosis of IDH-mutated low-grade gliomas (LGGs), these tumors are still regarded as incurable. Irrespective of the significant advancements in enhancing the extent of surgical removal and the efficacy of adjuvant radiochemotherapy, they frequently undergo malignant transformation **[14]**. Consequently, there is an urgent demand for innovative treatment approaches to address this issue. 

## 3. Challenges of Immunotherapy in LGG (Figure 1)

The development of novel immunotherapies presents challenges arising from the unique immunology and localization characteristics of solid tumors. As we have extensively examined the immunology of low-grade gliomas (LGG) just recently [14], in the subsequent discourse, we highlight the most significant challenges associated with immunotherapies in LGG.

(1) Gliomas, in general, are considered to have a “cold” immunosuppressive microenvironment. Compared to their malignant counterparts, IDH mutant LGG share a unique overall immunosuppressive immune phenotype that is characterized by a lower number of T cell infiltrations [15,16,17,18], a delayed recruitment of monocyte-derived macrophages [17,19], and inactive microglia [14,17]. This may be due to the following: intact blood–brain barrier (BBB) [20,21], high tumor purity with low mutational burden (TMB) [22], and altered energy metabolism [23]. Notably, low tumor mutational burden (TMB) is not exclusive to LGG and can also be observed in HGG. In the context of LGG, the lower mutational burden is highly likely associated with the comparatively slower growth rate of these tumors. Conversely, in the context of HGG, it has been suggested that lower TMB in recurrent glioblastoma may be indicative of neoantigen depletion through immunoediting [24,25].

In addition to the IDH-mutant immunosuppressive enzyme activity, kynurenine, a product of the enzyme tryptophan 2,3-dioxygenase (TDO), has been recently characterized to induce aryl hydrocarbon receptor (AhR) activation, thereby promoting immunosuppressive myeloid states [26]. Furthermore, extracellular vesicles derived from the IDH-mutant glioma have been demonstrated to induce local and systemic immunosuppression by decreasing the presence of effector lymphocytes such as natural killer (NK) cells and increasing the numbers of regulatory T (Treg) cells [27,28]. 

(2) Standard treatment can be immunosuppressive. Some studies have observed that the current treatment strategy with adjuvant RT chemotherapy further decreases immune cell infiltration, thereby hampering potential immunotherapeutic approaches [29,30]. In addition, the induction of lymphopenia, RT, and chemotherapy have been reported to increase the proportion of regulatory T cells that are considered to hamper the efficacy of immunotherapy [30]. Further research is required to assess the impact of adjuvant radiotherapy (RT) and chemotherapy on immunotherapy, as the implications of lymphopenia on the induction of effective immune responses remain uncertain. These investigations are of utmost importance to determine the optimal timing for administering immunotherapy. 

(3) Marked inter- and intra-patient heterogeneity of the disease. IDH-mutant LGG show a heterogeneous cell population consisting of differentiated and undifferentiated tumor cells derived from distinct origins [19,31], along with differences in morphology and heterogenous tumor antigen expression, thereby lacking common or unique tumor-specific antigens [18].

(4) Challenges to selecting patient populations. LGGs are relatively rare when compared to other tumor entities. The exact number of LGG is difficult to accurately determine because tumor registries have only recently started using the 2021 WHO CNS classification. Prior to this classification, studies indicated that the incidence was between about 0.51 and 0.25 per 100,000 per year for astrocytoma and oligodendroglioma [32,33]. Taking this into consideration, studies must be carefully designed to ensure sufficient statistical power for efficacy evaluation.

(5) Lack of accurate preclinical models. To facilitate the discovery of new biological insights and the development of novel therapeutics for low-grade gliomas (LGGs), it is essential to have preclinical models that faithfully recapitulate human disease. However, most preclinical studies have predominantly focused on high-grade gliomas (HGGs), as they are easier to maintain in rapidly growing cell cultures and consistently reproducible in murine models. In contrast, the development of preclinical models for LGGs has encountered challenges due to the slow-growing nature of these tumors and the complexities involved in reproducing the broad genomic and epigenomic effects of IDH mutation. In vitro generation of murine IDH-mutant cell lines or the establishment of stable patient-derived IDH-mutant lines that maintain the IDH-mutant status have remained elusive [34].

Consequently, the lack of well-characterized and representative preclinical models hampers the ability to effectively evaluate and validate potential therapeutic strategies, posing obstacles to the successful translation of promising preclinical discoveries into clinical applications.

## 4. Inhibitors of IDH Mutant Enzymatic Activities Can Reverse the Immunosuppressive Environment

Most (Around 90% of) IDH-mutated LGGs have a heterozygous point mutation in IDH1 that causes an arginine-to-histidine substitution at amino acid 132 (IDH1 R132H). This alteration leads to a gain-of-function mutation that prevents the conversion of isocitrate to alpha-ketoglutarate (α-KG) [23].

The result is the formation of the oncometabolite D-2-hydroxyglutarate (D-2HG) [35]. D-2HG structurally resembles α-KG and triggers glioma development in part by competitively inhibiting tumor suppressors in the α-KG-dependent dioxygenase family. In addition, mutant IDH enzyme activity alters cellular energy metabolism, such as the Krebs cycle. Mutant IDH enzymes also consume NADPH to produce D-2HG, reducing the availability of this redox cofactor for de novo lipogenesis and increasing dependence on exogenous lipids [36,37]. Interestingly, inhibition of IDH mutant enzymatic activity can influence the tumor microenvironment [38].

In the preclinical animal models, Kohanbash et al. demonstrated that the introduction of an IDH-1 mutation in immortalized normal human astrocytes and syngeneic mouse glioma models, or treatment with 2HG, led to a decrease in Signal transducer and activator of transcription 1 (STAT1), the master regulator of type I and type II interferon responses, leading to the inhibition of C-X-C motif chemokine ligand 10 (CXCL10). Consequently, there is decreased T-cell infiltration in the tumor. This effect was reversed by IDH-C35, a specific inhibitor of the IDH1 mutation. In addition, IDH inhibitors also improved the efficacy of vaccine immunotherapy against glioma-associated antigens (GAA) in mice expressing IDH-mutated tumors [39]. Chuntova et al. have further demonstrated that treatment of mice that harbor HLA-A2/HLA-DR1-syngeneic IDH1R132H tumor cells with the IDH mutant inhibitor (AG-881) suppressed the progression of IDH1R132H glioma. This effect was dependent on CD4+ and CD8+ T cell activity. Additionally, vaccination with both HLA-A2-IDH1R132H and DR1-IDH1R132H peptides in combination with either AG-881 or PD-1 blocking antibodies significantly prolonged survival [40].

Bunse et al. found that the oncometabolite, R-2-HG, is taken up by infiltrating T cells in the xenograft and syngeneic models. This interferes with the transcriptional activity of nuclear factor-activated T cells and polyamine biosynthesis, thereby hampering overall T cell activity [41]. The immunological clustering of human gliomas based on the enrichment levels of 28 immune cells in the tumor immune microenvironment highlighted that IDH mutations negatively correlated with glioma immunity [42]. Low glioma immunity was associated with lower tumor stemness and epithelial-mesenchymal transition scores, less tumor progression, lower mutational burden, and less frequent somatic copy number alterations [42].

Kadiyala et al. demonstrated that combination therapy of D-2HG inhibition with radiation and temozolomide led to higher median survival in mice that harbor mIDH1 gliomas. Moreover, the authors observed significant upregulation of PD-L1 expression in the glioma cells following this regimen, so they added anti-PDL-1 immune checkpoint blockade to the therapy, resulting in complete tumor regression in 60% of animals bearing mIDH1 glioma. Interestingly, the therapeutic success was attributed to reduced T cell exhaustion and the generation of CD8+ memory T cells [43].

In 2021, Platten et al. presented an exciting multicenter, single-arm, open-label, first-in-humans phase I vaccine trial carried out in 33 patients with newly diagnosed WHO grade 3 and 4 IDH1(R132H)+ astrocytomas. In this trial, an IDH1(R132H)-specific peptide vaccine promoted immune responses in 93.3% of patients across multiple MHC alleles, and patients with immune responses showed a two-year progression-free rate of 0.82. Two patients without an immune response showed tumor progression within two years of the initial diagnosis. Combined single-cell RNA and T cell receptor sequencing revealed that tumor-infiltrating T helper cell clusters in a patient with pseudoprogression were dominated by a single IDH1(R132H)-reactive T cell receptor. Although this study was performed in IDH-mutant WHO grade III and IV glioma patients, it offers a promising perspective for future vaccine trials in IDH-mutant low-grade glioma [44].

These positive results highlight that IDH inhibition may represent a promising future treatment for IDH-mutated gliomas. In line with this, the US Food and Drug Administration (FDA) has recently granted fast track designation to Vorasidenib (AG-881), a novel IDH-inhibitor that showed promising results in the multicenter, randomized, double-blind clinical trial INDIGO [45,46]. In this trial, vorasidenib treatment resulted in low toxicity, prolonged progression-free survival (PFS), and delayed time to next intervention (TTNI) in patients with residual or recurrent IDH-mutant low-grade gliomas whose only prior treatment was surgery [47].

Beyond mutant IDH inhibition, recent studies have identified additional targetable mechanisms for immunosuppression in LGGs. Abdelfattah N. et al. identified S100A4 as a regulator of immune suppressive T and myeloid cells in both HGGs and LGGs. In their study, the deletion of S100A4 in non-cancer cells was sufficient to reprogram the immune landscape and significantly improve survival [48]. Likewise, Tao B. et al. revealed a novel role for CYB561D2 in mediating the crosstalk between reactive oxygen species and tumor immunity. They demonstrated that CYB561D2 upregulation leads to immunosuppression and activation of STAT3 in gliomas [49]. Furthermore, Alghamri et al. characterized G-CSF in an interesting preclinical study as a potential modulator of bone marrow granulopoiesis, leading to the generation of non-inhibitory myeloid cells that counteract the immunosuppressive environment [50].

Beyond that, the application of oncolytic viruses holds promise for glioma therapy in the future. Following the approval of the oncolytic herpesvirus T-vec for melanoma treatment, numerous experimental virotherapies have entered clinical studies for various tumor types, including malignant gliomas [51]. Encouragingly, results from certain clinical trials investigating oncolytic viruses (OVs) in malignant glioma patients have demonstrated the ability of viruses to infect tumor cells and enhance immune cell recruitment, despite the immunosuppressive tumor microenvironment (TME) [52,53,54,55,56]. Nevertheless, no studies have been conducted thus far investigating the use of oncolytic viruses, specifically in the context of IDH-mutant gliomas.

In addition to that, checkpoint inhibitors have emerged as an additional approach to counteract the immunosuppressive environment, which is critical for maintaining self-tolerance and regulating immune responses to minimize tissue damage in peripheral tissues [57]. Within the context of tumor cells, myeloid-derived suppressor cells are stimulated to upregulate programmed death ligand 1 (PD-L1) on their cell surface, engaging in immune checkpoint modulation that impacts T cell responses [57,58,59]. In normal circumstances, PD-L1 binds to programmed cell death protein 1 (PD-1) on T cells, resulting in the suppression of T cell activation and the prevention of autoimmune diseases. However, in tumors, the interaction between PD-L1 on tumor cells and PD-1 on killer CD8+ T cells impedes the recognition and elimination of tumor cells, favoring tumor survival and progression. Interestingly, in the context of gliomas, it has been observed that the presence of an isocitrate dehydrogenase (IDH) mutation is associated with reduced expression of immunological checkpoint molecules, including PD-1, cytotoxic T-lymphocyte-associated protein 4 (CTLA-4), lymphocyte-activation gene 3 (LAG3), and indoleamine 2,3-dioxygenase 1 (IDO1) [58]. IDH-mutant astrocytomas exhibit a comparatively higher responsiveness to checkpoint immunotherapy compared to IDH-mutant oligodendrogliomas, primarily due to the reduced expression of PD-L1 and other checkpoint molecules in the latter. Moreover, higher levels of T cell exclusion contribute to T cell dysfunction and resistance to immunotherapy in IDH-mutant oligodendrogliomas [59,60,61]. Consequently, considering the lower expression of PD-L1 in IDH-mutant patients, inhibitors targeting the PD-1/PD-L1 immune checkpoint may not be ideal, and alternative therapeutic options should be investigated.

## 5. Development of Vaccines

Novel vaccine strategies have gained popularity and raised hopes for improving the treatment of various cancers, even prior to the COVID-19 pandemic. The main goal of these vaccines is to harness the power of endogenous immune cells to recognize and attack cancer cells. Several vaccine approaches are being investigated mainly in high-grade gliomas, including peptide-based vaccines, dendritic cell vaccines, viral vector vaccines, and personalized neoantigen vaccines, but recently a few studies have investigated the efficacy of vaccines targeting LGG. Each approach has its own strengths and limitations, but collectively, they represent a concerted effort to combat glioma through immunotherapy. As IDH-mutant LGG show a relatively “cold” immunological environment, immune adjuvants may be necessary to enhance the activity of migrated T cells. In this context, polyinosinic:polycytidylic acid, or poly(I:C), and its derivative poly-ICLC have been characterized as potential supportive therapies for priming or boosting lymphocytes and other factors in the (immuno) therapeutic regimen against glioma [62]. 

Poly(I:C) and poly-ICLC are synthetic double-stranded RNA molecules (dsRNA) that are comprised of a polyinosinicacid homopolymer annealed to a polycytidylic acid homopolymer that builds together a stable double helix [63]. Both interact with endosomal Toll-like receptor (TLR)-3 as well as with the cytoplasmic receptors, retinoic acid-inducible gene I (RIG-I), and melanoma differentiation-associated gene 5 (MDA-5), thereby mimicking a viral infection in inducing the secretion of type I interferon (IFN) and pro-inflammatory cytokines by antigen-presenting cells. Clinically, poly-ICLC has been used mostly as an adjuvant to dendritic cell or peptide vaccines, where it has been demonstrated to be safe and capable of eliciting immunological activity to boost therapeutic responses [62,64,65,66,67,68]. 

In 2015, we reported the first cancer vaccine study specifically designed for patients with WHO Grade 2 LGG that uses glioma-associated antigen (GAA)-derived epitopes and adjuvant poly-ICLC [68]. In this three-arm phase I study, 33 patients were stratified into three cohorts: 1: patients without prior progression, chemotherapy, or radiotherapy (RT); 2: patients without prior progression or chemotherapy but with prior RT; and 3: recurrent patients. GAA Interleukin-13 receptor α2 (IL13Rα2), Ephrin type-A receptor 2 (EphA2), Wilms’ tumor gene 1 (WT1), and survivin were chosen as they are expressed in both high- and low-grade gliomas. Synthetic peptides were emulsified in Montanide-ISA-51 and given every 3 weeks for eight courses with intramuscular injections of poly-ICLC, followed by q12-week booster vaccines [68]. The vaccine was well tolerated, and immune reactivity was observed in the majority of patients [68]. Patients in Cohort 1 presented significantly higher levels of vaccine-reactive T cell responses than those in Cohorts 2 and 3. 

Pollack et al. initiated another pilot trial of vaccinations with peptides for GAA epitopes in HLA-A2+ children with recurrent LGG that had progressed after at least two prior regimens. In this study, peptide epitopes for 3 GAAs (EphA2, IL-13Rα2, and survivin) were emulsified in Montanide-ISA-51 and administered subcutaneously adjacent to intramuscular injections of polyinosinic-polycytidylic acid stabilized by lysine and carboxymethylcellulose (poly-ICLC) every 3 weeks for 8 courses, followed by booster vaccines every 6 weeks. Vaccination-induced immunoreactivity to at least one vaccine-targeted GAA in all 12 patients [65]. Although these pediatric LGG tumors did not harbor IDH-mutant enzymes, this study underscores the impression that the immunologically “cold” LGG could be receptive to vaccine approaches. 

In 2022, Ogino and Tayler et al. conducted a randomized trial of neoadjuvant vaccination with lysate derived from an allogeneic glioblastoma stem cell line (GBM6-AD) with poly-ICLC in patients with WHO grade II LGGs [69]. Patients were randomized to receive the vaccines (arm 1) or not (arm 2) before clinically indicated surgery, and all patients received adjuvant vaccines. The vaccine was well tolerated and activated CD8+ T cell clones, some of which were detected in the tumor microenvironment. However, the upregulation of cytokines and chemokines detected in the peripheral blood was not detected in the CNS tumor tissue, in contrast to the previous GBM trial [66]. 

The studies highlighted a crucial take-home message: immune responses have been observed in vaccination studies without significant adverse effects. However, the efficacy of vaccination alone in fighting tumors remains limited. A critical factor impeding the success of vaccination therapies is the immunological privilege of the brain, which poses challenges in mobilizing sufficient numbers of antigen-specific cells into the tumor. Overcoming this hurdle holds the key to unlocking the full potential of vaccination as a promising therapeutic approach, particularly in the younger patient population. Further research and advancements in strategies to surmount the brain’s immunological barriers are needed to maximize the effectiveness of vaccination in the treatment of brain tumors.

## 6. Strategies to Address the Immune Privilege of the Brain to Enhance Immune Cell Migration and Drug Delivery to the Brain

**Figure 1:**
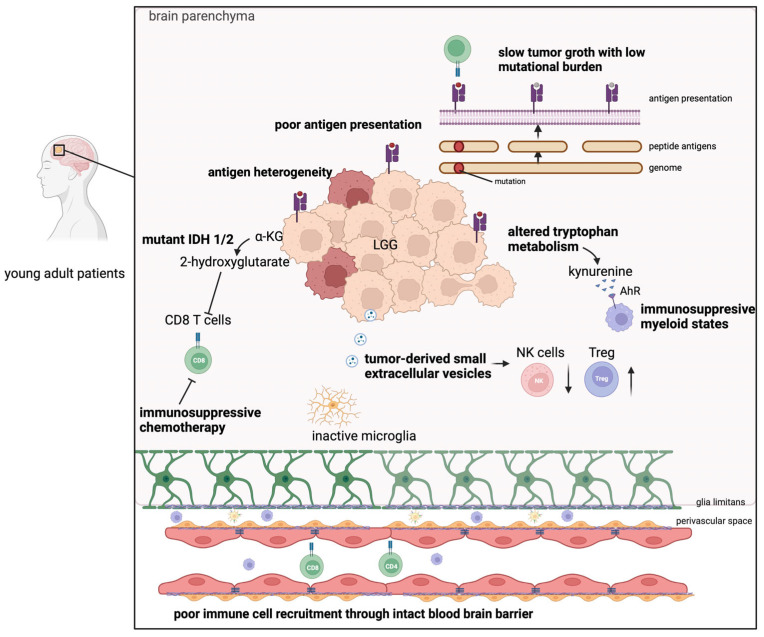
Challenges for immunotherapy in IDH-mutant low-grade gliomas. LGG are immunologically “cold” and poorly infiltrated by immune cells due to the following reasons: They usually have an intact blood-brain barrier that is composed of the endothelial cells, their basement membrane, the perivascular space, and the astrocyte endfeet that form the glia limitans and hinders homing of the immune cells into the brain parenchyma. LGG have mutant IDH 1/2 enzymes leading to the generation of 2-hydroxygluatrate, which suppresses (⊥) T cell activity. Additional alterations in the tryptophan metabolism have been identified, inducing immunosuppressive myeloid states via kynurenine. Furthermore, tumor-derived small extracellular vesicles have been suggested to inhibit effector T cell and NK cell activity (↓) and increase the infiltration of Tregs (↑). Beyond that, preclinical studies have observed that IDH-mutation is associated with less immunoreactive microglia. However, the exact mechanism must be determined. Moreover, while LGG have a low mutational burden, a high level of inter- and intra-patient heterogeneity has been observed, making it challenging to design antigen-specific therapies.

Over a century ago, it was demonstrated that the CNS parenchyma is immunologically privileged compared to the rest of the body due to the anatomical barriers that hinder immune cells from entering and leaving the CNS parenchyma [70,71]. One of these natural barriers is the BBB, formed by the endothelial cells of the capillaries, their basement membrane, the perivascular space, and the glia limitans (Figure 1). The latter is a final barrier formed by the projections of astrocytes, which immune cells must overcome to migrate from the CSF-filled perivascular space into the parenchyma [70,71,72]. New technological approaches, such as low-intensity pulsed focused ultrasound combined with the application of microbubbles, now offer a way to reversibly open this otherwise closed barrier for a short period, thereby allowing immune cell immigration or drug transport into the brain [73,74,75,76,77]. These microbubbles are selectively oscillated in the bloodstream by MRI-targeted low-intensity focused ultrasound waves and can thus open tight junctions, which connect the endothelial cells tightly together like a belt [73,74]. Initial preclinical and clinical studies in the field of malignant gliomas have shown improved infiltration of immune cells into the tumor [75,76], and recently Sonabend et al. demonstrated in an interesting phase-1 trial where patients received a skull-implantable ultrasound device that repeated sonication treatment is safe and allows repeated penetration of the albumin-bound chemotherapeutic drug paclitaxel into the brain [78]. While malignant tumors usually already show some BBB disruption in certain areas [20], it appears intuitive that a combination of focused ultrasound with immunotherapies might be even more effective in LGG, which are characterized by a generally intact BBB [21]. 

Beyond that, recent studies have discussed the glioma extracellular matrix (ECM) as a potential additional physical barrier in immunotherapy [79,80,81]. The ECM serves as a biochemical and biophysical scaffold for the cellular components within the TME. It comprises various components, such as interstitial fluid, minerals, and fibrous proteins, including collagen and elastin, which provide structural integrity and tensile strength. Additionally, adhesive glycoproteins such as fibronectin, laminin, and tenascin play a role in cell adhesion. The non-fibrillar constituents of the ECM include proteoglycans such as heparan sulfate, chondroitin sulfate, and keratan sulfate, as well as glycosaminoglycans like hyaluronic acid. Collectively, these components contribute to the composition and functionality of the ECM within the tumor microenvironment [79]. The motility and infiltration of lymphocytes are influenced by their interactions with the extracellular matrix (ECM). Current knowledge suggests a correlation between the aggressiveness of adult gliomas, patient prognosis, and the stiffness of the extracellular matrix (ECM) [82]. Generally, non-tumor tissue exhibits the lowest level of ECM rigidity, while LGG and glioblastoma multiforme (GBM) exhibit higher rigidity [83]. In areas where the ECM fibers are densely packed, there is a reduced abundance of tumor-infiltrating T cells [84]. The targeting of the extracellular matrix (ECM) through various strategies such as antibodies or ligands, RNA interference, pharmacological agents, and modification of ECM molecules, as recently reviewed by Mohiuddin et al. [85], represents a potential avenue to enhance the penetration and efficacy of immune cell therapy. 

Beyond that, locoregional delivery such as intraventricular (i.c.v.), intracavitary (i.c.), or intratumor (i.t.) immunotherapy application has to be investigated in the context of IDH-mutant low-grade glioma in the future, as it has been demonstrated in several human phase I HGG trials to be a potential alternative to overcome the natural barriers [86,87,88]. These application routes may allow for lower drug concentrations and reduce the risk of off-target toxicities. 

## 7. Antigen Heterogeneity and Potential Mitigation Strategies

HGG are widely recognized for their prominent intra-tumoral genetic and immunological heterogeneity [89,90]. However, it is important to note that IDH-mutant low-grade gliomas (LGGs) have also been reported to exhibit a considerable degree of heterogeneity. On an evolutionary basis, single-cell RNA sequencing (scRNA-seq) performed by Tirosh et al. on six IDH-mutant oligodendrogliomas identified distinct copy number variant sub-clones within tumors, demonstrating that the branched genetic evolution of oligodendroglioma cells is a crucial determinant of tumor heterogeneity. More striking is their finding that non-genetic programs, such as those associated with the self-renewal of tissue stem cells and their differentiation into specialized cell types, contribute further to tumor functional heterogeneity [31]. Murine models also demonstrate that genomic heterogeneity is progressively acquired in LGG progression into higher grades [91]. This is consistent with findings from Mazor T. et al., where methylation profiling of spatially distinct pieces of the same tumor tissues revealed subtle intratumoral divergences, which were exasperated upon recurrence. Their clonal evolution model highlights that intratumoral heterogeneity extends to epigenetic alterations. After surgical resection, LGGs continue to evolve subclones with genetic and co-dependent epigenetic features distinct from the initial tumor [92]. Furthermore, a combination of targeted therapies and immunotherapies may be necessary to address the heterogeneity and evolution of low-grade gliomas, including the potential emergence of treatment-resistant subclones.

Similarly, intratumoral heterogeneity within IDH-mutant gliomas extends to immune cell populations across the tumor microenvironment. Tumor-associated macrophages (TAMs) comprise the majority of immune cells within gliomas, with studies demonstrating that TAMs within IDH-mutant cases are predominantly microglia [16,17]. With scRNA-seq analysis performed by Venteicher A et al., they unveiled significantly different microglia and microphage intratumoral compositions between astrocytoma and oligodendroglioma subtypes [19]. Amongst other infiltrating immune cell types, it is widely known that the IDH mutation leads to drastically altered tumor immunology, notably decreased CD8 and CD4 T-cell numbers and infiltration into the tumor tissue compared to IDH-wild type cases [18,39,41]. We previously analyzed the T-cell repertoire across multiple biopsies performed on approximately ten intratumorally spatially mapped sites within LGGs and identified T-cell clones targeting patient-specific neoantigens infiltrating multiple intratumoral sites and peripheral blood.

Similarly, many of these somatic mutation-derived neoantigens were confirmed to be expressed tumor-wide in all spatially mapped sites [93]. While the heterogeneity of the LGG landscape has been challenging to navigate, these findings obtained with a higher depth of spatial analysis demonstrate that tumor-specific T-cells do infiltrate multiple compartments of the LGG environment to target intratumorally conserved neoantigens. Many approaches can be taken to tackle the issue of intratumoral heterogeneity in LGGs. One is T-cell-based immunotherapy targeting multiple tumor-specific antigens, otherwise known as neoantigens. Single-antigen targeting often leads to incomplete tumor eradication, resulting in recurrence by antigen-negative cells. Combining multiple single-antigen approaches could yield multi-focal strategies for tackling mutational divergence in IDH-mut gliomas. Most notably, we have identified public tumor-wide splicing-derived neoantigen targets in both LGG and GBM cases. We validated these targets on a transcriptomic and proteomic level and also proved that they elicit a T-cell-mediated immune response. More importantly, our study performed spatial transcriptomics on at least 10 maximally-distanced biopsies across 56 glioma patients, and we concluded that these novel neoantigen candidates were expressed tumor-wide. TCR-transduced T-cells demonstrated proficient recognition of these splicing-derived peptides, and their results illustrate a novel repertoire of heterogenous neoantigen targets for gliomas [93]. In a separate study, we demonstrated a streamlined approach for identifying personalized tumor-specific T-cell clones in the peripheral blood of WHO Grade II astrocytoma patients. Similar spatial analysis of multiple biopsies revealed that the neoantigen targets of these T-cell clones were also products of tumor-wide truncal mutations [93]. 

Another strategy could focus on identifying and targeting stem cell-like populations within tumors to slow progression. Tirosh I. et al.’s scRNA-seq results illustrate a cancer stem cell model in which the most primitive and undifferentiated population of cancer cells serves as the primary source of proliferating cells in oligodendrogliomas [31]. Similar scRNA-seq studies conducted by Gojo J. et al. identified the enrichment of undifferentiated cell subpopulations within ependymoma, which can inform treatment strategies to promote differentiation [94]. Immunotherapies targeting glioma stem cell (GSC) markers are underway for HGGs. For example, CD133-specific chimeric antigen receptor (CAR)-T cells developed by Vora et al. have demonstrated preclinical success in targeting self-renewing and chemoradioresistant CD133+ brain tumor-initiating cells [95]. With a notable expression of CD133 in low-grade glial progenitors, this also opens the potential for GSC-targeting CAR T-cell approaches to be translated into LGG applications [96]. Although targeting the progenitor cell derivatives via their intrinsic biomarkers can be a promising strategy for treating LGGs, more investigations are needed to determine the toxicity and side effects of such an approach, as normal human hematopoietic stem cells are also known to express CD133 [97]. In general, CAR therapies targeting gliomas have shown promise, particularly in the context of HGGs [86,87,88]. Some of these therapeutic approaches may also hold potential for IDH-mutant LGGs, despite the relatively low expression of target antigens in IDH-mutant gliomas. However, the future success of CAR therapy for LGGs depends on the identification of antigens that are specific to LGGs. 

## 8. Novel Biomarkers to Non-Invasively Assess the Efficacy of Immunotherapy in LGG Are Needed

As immunotherapies can cause inflammatory responses, thereby increasing the size of the pathological mass, the interpretation of tumor imaging has become challenging due to pseudoprogression. This phenomenon refers to the temporary appearance of tumor growth, even though the actual tumor size may not have increased [98,99]. There are two primary explanations for a potential discrepancy between the initial worsening of imaging results and subsequent therapeutic benefits. Firstly, effective immune responses require time to develop, and the initial imaging could indeed indicate genuine disease progression. However, once an effective immune response is triggered, it could ultimately lead to clinical improvements. Secondly, since the treatment’s mechanism of action may involve an inflammatory response in regions affected by visible and microscopic infiltrative tumors, localized inflammatory reactions might imitate radiographic characteristics of tumor progression, such as contrast enhancement and edema [100]. Therefore, a multidisciplinary panel of neuro-oncology immunotherapy experts has established the immunotherapy response assessment for neuro-oncology (iRANO) criteria, allowing us to better distinguish between pseudoprogression and actual tumor growth. To avoid the false assumption of pseudoprogression, these criteria recommend performing follow-up imaging not earlier than 3 months after initial radiographic progression if the patient shows no (substantial) clinical deterioration [98]. Although these novel guidelines helped to address the phenomenon of pseudoprogression, novel biomarkers that non-invasively assess the efficacy of immunotherapy in LGG are urgently needed. Novel imaging approaches, such as quantitative evaluation of 2-hydroxyglutarate levels by magnetic resonance spectroscopy, are currently being investigated in the clinical trial NCT03952598 [101,102]. Further development of these approaches will help assess LGG tumor remnants vs. progression.

## 9. Conclusions

Recent preclinical and clinical trials have highlighted that immunotherapy for LGG may be a promising approach, particularly in young LGG patients that underwent gross total resection, allowing them to mount immune responses against critical drivers of high-grade transformation. Vaccine-reactive T cell responses were detected in the post-vaccine LGG tumor tissue, but homing of immune cells through the intact BBB remains challenging. Penetration of immune effectors could be achieved with novel technologies, such as low-intensity focused ultrasound combined with microbubbles, allowing targeted transient opening of the BBB in the tumor. Moreover, excellent studies have revealed the significant role of IDH mutations in the LGG immune environment and support the rationale for modulating the LGG by targeting the immunosuppressive IDH enzymatic activities. Furthermore, despite the marked genetic and antigenic heterogeneity, novel T cell-based approaches targeting neojunctions could lead to both personal and shared therapeutic approaches. 

## Figures and Tables

**Table 1:** Clinical trials investigating immunotherapy for low-grade glioma.

Children /Adults	Study Phase	ClinicalTrials.gov Identifier	Experimental Treatment	Cohort Size	Primary Endpoint/Outcomes	Results for Primary Outcome	Study Start	Current Status
IDH-Inhibitor								
Adults	Phase 1	NCT03343197	AG-120 (Ivosidenib), AG881 (Vorasidenib)	49	2HG concentration in resected tumors	Decreased tumor cell proliferation and immune cell activation	March 2018	Active, not recruiting
Adults	Phase 1	NCT03030066	DS-1001b	47	Percentage of participants with dose-limiting toxicities	No dose-limiting toxicities	January 2017	Active, not recruiting
Adults	Phase 1	NCT04762602	HMPL-306	90	Treatment emergent adverse events (TEAEs), dose-limiting toxicities	Not yet posted for glioma patients that were included	February 2021	Recruiting
Adults	Phase 2	NCT04056910	Ivosidenib +Nivolumab	35	6-month progression-free survival, best overall response (time frame: 8 weeks–14 months)	Not yet posted	September 2021	Active, not recruiting
Adults	Phase 2	NCT04458272	DS-1001b	25	Objective Response Rate: complete response (CR) + partial response (PR), number of patients with TEAEs	Not yet posted	July 2020	Active, not recruiting
Adults	Phase 2	NCT05303519	Safusidenib	95	TEAEs, proportion of patients with the best overall confirmed response of CR or PR	Not yet posted	May 2023	Recruiting
Children, Adults	Phase 3	NCT04164901	Vorasidenib	331	Progression-free survival	Significantly higher PFS in the AG-881 group (27.7 months vs. 11.1 months)	January 2020	Active, not recruiting
Vaccines/immune-adjuvants								
Adults	Phase 1	NCT02924038	IMA950, poly-ICLC, varlilumab	14	Incidence of AEs, evaluation of CD4/CD8+ T cell response	Well-tolerated, vaccine-reactive T-cell expansion in the peripheral blood, but not in the tumor	April 2017	Active, not recruiting
Children, Adults	Phase 1	NCT01130077	HLA-A2-restricted glioma antigen peptide vaccine, poly-ICLC	60	Safety	No dose-limiting non-CNS toxicity, 21 of 26 children showed positive anti-GAA immune responses	February 2009	Active, not recruiting
Adults	Phase 1	NCT00795457	GAA/TT-peptide vaccine and poly-ICLC	13	Induction of GAA-specific T-cell response and safety	Well tolerated, robust-GAA-specific responses	January 2009	Completed
Adults	Phase 1	NCT02549833	GBM6-AD, poly-ICLC	28	Toxicity, immune response in the tumor	No dose-limiting toxicity, effector CD8 T-cell response in blood and tumor microenvironment	October 2016	Active, not recruiting
Adults	Phase 1	NCT05609994	PEPIDH1M vaccine in combination + Vorasidenib	48	Proportion of patients with unacceptable toxicity, progression-free survival	Not yet posted	Estimated: July 2023	Not yet recruiting
Adults	Phase 2	NCT01635283	Tumor lysate pulsed autologous dendritic cell vaccine	5	Progression-free survival (up to 44 months)	Time without being affected by tumor recurrence or progression: >30 months (n = 2/5)	January 2012	Completed
Children, Adults	Phase 2	NCT02358187	HLA-A2 Restricted Glioma Antigen-Peptides with Poly-ICLC	25	Tumor shrinkage or stable disease	Not yet posted	January 2015	Recruiting
Children, Adults	Phase 2	NCT04544007	Poly-ICLC	20	Objective Response Rate (PR + CR)	Not yet posted	December 2021	Recruiting
Children, Adults	Phase 2	NCT01188096	Poly-ICLC	23	Objective Response Rate (PR + CR)	43% stable disease, 17% partial responses	August 2010	Completed
PD-1 Inhibition								
Adults	Phase 2	NCT03718767	Nivolumab	70	6-month progression-free survival	Not posted yet	March 2019	Recruiting
Adults	Phase 2	NCT03557359	Nivolumab	20	Objective Response Rate (PR + CR)	Not posted yet	June 2018	Active, not recruiting
