# Correction: Gallus et al. Immunotherapy Approaches in Isocitrate-Dehydrogenase-Mutant Low-Grade Glioma. *Cancers* 2023, *15*, 3726

**DOI:** 10.3390/cancers16010119

**Published:** 2023-12-26

**Authors:** Marco Gallus, Darwin Kwok, Senthilnath Lakshmanachetty, Akane Yamamichi, Hideho Okada

**Affiliations:** 1 Department of Neurological Surgery, University of California, San Francisco, CA 94143, USA; 2 Department of Neurosurgery, University Hospital Muenster, 48149 Muenster, Germany; 3 Parker Institute for Cancer Immunotherapy, San Francisco, CA 94129, USA; 4 Helen Diller Family Comprehensive Cancer Center, San Francisco, CA 94143, USA

## Correction of Table 1

It has come to our attention that the previously published manuscript contained an outdated iteration of Table 1 [1].

The revised version incorporates the accurate table encompassing all pertinent studies with the appropriate NCT identifiers. Additionally, we have restructured the arrangement of studies, commencing with phase I and progressing to later-phase trials.

The authors apologize for any inconvenience caused and state that the changes do not affect the scientific results. This correction was approved by the Academic Editor. The original publication has also been updated.

## Figures and Tables

**Table 1:** Clinical trials investigating immunotherapy for low-grade glioma.

Children/Adults	Study Phase	ClinicalTrials.gov Identifier	Experimental Treatment	Cohort Size	Primary Endpoint/Outcomes	Results for Primary Outcome	Study Start	Current Status
IDH-Inhibitor								
Adults	Phase 1	NCT03343197	AG-120 (Ivosidenib), AG881 (Vorasidenib)	49	2HG concentration in resected tumors	Decreased tumor cell proliferation and immune cell activation	March 2018	Active, not recruiting
Adults	Phase 1	NCT03030066	DS-1001b	47	Percentage of participants with dose-limiting toxicities	No dose-limiting toxicities	January 2017	Active, not recruiting
Adults	Phase 1	NCT04762602	HMPL-306	90	Treatment emergent adverse events (TEAEs), dose-limiting toxicities	Not yet posted for glioma patients that were included	February 2021	Recruiting
Adults	Phase 2	NCT04056910	Ivosidenib + Nivolumab	35	6-month progression-free survival, best overall response (time frame: 8 weeks–14 months)	Not yet posted	September 2021	Active, not recruiting
Adults	Phase 2	NCT04458272	DS-1001b	25	Objective Response Rate: complete response (CR) + partial response (PR), number of patients with TEAEs	Not yet posted	July 2020	Active, not recruiting
Adults	Phase 2	NCT05303519	Safusidenib	95	TEAEs, proportion of patients with the best overall confirmed response of CR or PR	Not yet posted	May 2023	Recruiting
Children, Adults	Phase 3	NCT04164901	Vorasidenib	331	Progression-free survival	Significantly higher PFS in the AG-881 group (27.7 months vs. 11.1 months)	January 2020	Active, not recruiting
Vaccines/immune-adjuvants								
Adults	Phase 1	NCT02924038	IMA950, poly-ICLC, varlilumab	14	Incidence of AEs, evaluation of CD4/CD8+ T cell response	Well-tolerated, vaccine-reactive T-cell expansion in the peripheral blood, but not in the tumor	April 2017	Active, not recruiting
Children, Adults	Phase 1	NCT01130077	HLA-A2-restricted glioma antigen peptide vaccine, poly-ICLC	60	Safety	No dose-limiting non-CNS toxicity, 21 of 26 children showed positive anti-GAA immune responses	February 2009	Active, not recruiting
Adults	Phase 1	NCT00795457	GAA/TT-peptide vaccine and poly-ICLC	13	Induction of GAA-specific T-cell response and safety	Well tolerated, robust-GAA-specific responses	January 2009	Completed
Adults	Phase 1	NCT02549833	GBM6-AD, poly-ICLC	28	Toxicity, immune response in the tumor	No dose-limiting toxicity, effector CD8 T-cell response in blood and tumor microenvironment	October 2016	Active, not recruiting
Adults	Phase 1	NCT05609994	PEPIDH1M vaccine in combination + Vorasidenib	48	Proportion of patients with unacceptable toxicity, progression-free survival	Not yet posted	Estimated: July 2023	Not yet recruiting
Adults	Phase 2	NCT01635283	Tumor lysate pulsed autologous dendritic cell vaccine	5	Progression-free survival (up to 44 months)	Time without being affected by tumor recurrence or progression: >30 months (n = 2/5)	January 2012	Completed
Children, Adults	Phase 2	NCT02358187	HLA-A2 Restricted Glioma Antigen-Peptides with Poly-ICLC	25	Tumor shrinkage or stable disease	Not yet posted	January 2015	Recruiting
Children, Adults	Phase 2	NCT04544007	Poly-ICLC	20	Objective Response Rate (PR + CR)	Not yet posted	December 2021	Recruiting
Children, Adults	Phase 2	NCT01188096	Poly-ICLC	23	Objective Response Rate (PR + CR)	43% stable disease, 17% partial responses	August 2010	Completed
PD-1 Inhibition								
Adults	Phase 2	NCT03718767	Nivolumab	70	6-month progression-free survival	Not posted yet	March 2019	Recruiting
Adults	Phase 2	NCT03557359	Nivolumab	20	Objective Response Rate (PR + CR)	Not posted yet	June 2018	Active, not recruiting
